# The Implementation of Faricimab in East Lancashire NHS Trust: Highlighting the Real-World Efficacy and Safety in Patients With Neovascular Age-Related Macular Degeneration

**DOI:** 10.7759/cureus.93478

**Published:** 2025-09-29

**Authors:** Aimee Lloyd, Mahmoud Elbalawi, Miguel Kurc, Mariam Hussain, Salwa Abugreen

**Affiliations:** 1 Ophthalmology, Manchester Royal Eye Hospital, Manchester, GBR; 2 Ophthalmology, East Lancashire Hospitals NHS Trust, Blackburn, GBR; 3 Ophthalmology, University of Central Lancashire, Preston, GBR; 4 Ophthalmology, Hull York Medical School, Hull, GBR; 5 Ophthalmology, East Lancashire Teaching Hospital, Blackburn, GBR

**Keywords:** faricimab, intravitreal (ivt) faricimab, neovascular age-related macular degeneration, neovascular age-related macular disorders, real-world evidence

## Abstract

Purpose: To report the safety and efficacy of intravitreal faricimab injections (IVTF) in both treatment-naïve and treatment-resistant patients with neovascular age-related macular degeneration (nAMD).

Patients and methods: This study was a retrospective real-world evidence trial where patients with nAMD were given IVTF. Group 1 (treatment-naïve) and group 2 (switch loading dose, SLD) were given a course of four loading doses of IVTF four weeks apart. Group 3 (switch pro re nata, SPRN) was given a single IVTF and then reviewed at eight weeks. The outcome was based on visual acuity (VA) and optical coherence tomography (OCT), showing central retinal thickness (CRT), subretinal, and intraretinal fluid.

Results: A total of 99 eyes from 89 patients were included. A total of 350 IVTF were given. The mean follow-up duration was 6.33 ± 1.11 months (range: 2-8 months). The mean change in CRT at final follow-up was -127.09 ± 97.39 µm (p < 0.001) for naïve eyes, -115.59 ± 145.02 µm (p < 0.001) for SLD, and -57.61 ± 58.06 µm (p < 0.001) for SPRN. The mean VA change was -3.47 ETDRS (Early Treatment Diabetic Retinopathy Study) (p = 0.256) for naïve eyes, -0.79 (p = 0.45) for SLD, and +1.25 (p = 0.304) for SPRN. A total of 48 eyes (48.48%) had better VA, 22 (22.22%) had no change in VA, and 29 (29.29%) had worse VA. One patient developed bilateral sterile intraocular inflammation.

Conclusion: IVTF was associated with an improvement in CRT in both treatment-naive and treatment-resistant nAMD patients. There was no evidence of vasculitis or vein occlusion, highlighting the safety of faricimab in our real-world study.

## Introduction

Neovascular age-related macular degeneration (nAMD) is characterised by the growth of new abnormal vasculature, otherwise known as choroidal neovascularisations (CNV) [1]. The growth of CNV is stimulated by increased levels of vascular endothelial growth factor (VEGF) [2]. Vision loss can result from the development of exudation and fluid accumulation, which, if not promptly treated, can lead to subretinal fibrosis and retinal pigment epithelium (RPE) atrophy [1].

In the United Kingdom, several anti-VEGF drugs are licensed for use in nAMD, including ranibizumab and aflibercept [3]. Recently, brolucizumab was removed from use due to intraocular inflammation [4]. Patients typically receive treatment with anti-VEGF drugs on average every six weeks, with injection intervals ranging from four to 12 weeks [3]. This places a significant burden on the National Health Service (NHS) due to appointment demand [2]. In November 2022, the National Institute for Health and Care Excellence (NICE) approved a new drug called faricimab for treating nAMD [4].

Faricimab is the first licensed dual-action monoclonal antibody [5]. It is a humanised bispecific immunoglobulin G1 (IgG1) antibody that acts through inhibition of two independent pathways. The first pathway is like other anti-VEGF drugs, which help neutralise VEGF-A5. VEGF-A leads to vascular leakage and neovascularisation [5]. The second pathway is a novel target in nAMD treatment where faricimab neutralises angiopoietin-2 (Ang-2) [6]. It is thought that by acting on Ang-2, faricimab may help reduce the amount of fibrosis we see in nAMD [7].

Faricimab is manufactured by Roche© (Basel, Switzerland), and the manufacturer has carried out two large phase III trials (TENAYA and LUCERNE) [6,7]. The recommended loading dose for treatment-naïve patients is 6 mg (0.05 mL solution), administered every four weeks for the first four doses. Thereafter, treatment may be individualised using a treat-and-extend approach [6,7]. Here, we report the efficacy and safety of faricimab in both treatment-naïve and treatment-resistant patients from East Lancashire Hospitals NHS Trust (ELHT).

Purpose

The primary aim of the study was to collect real-world data from the ELHT on the efficacy and safety of faricimab in both treatment-naïve and treatment-resistant patients diagnosed with nAMD. Treatment-naïve patients are those who have never been treated with anti-VEGF drugs. Treatment-resistant patients had already received one or more anti-VEGF treatments (brolucizumab, ranibizumab, or aflibercept) in the past. ELHT has developed individual protocols for treatment-naïve (Appendix 1) and treatment-resistant (Appendix 2) patients, respectively. The secondary objective of this study was to share our method of faricimab implementation in a United Kingdom teaching hospital.

## Materials and methods

Participants

Patients were recruited from ELHT between 1st November 2022 and 27th February 2023, with the latest patient follow-up being on 30th July 2023. Inclusion criteria for this study were all patients diagnosed with nAMD receiving faricimab treatment. Both treatment-naïve (N = 30) and treatment-resistant (N = 60) eyes were included. The reason for switching a patient from an alternative anti-VEGF to faricimab was primarily due to persistent disease (92%) or at the discretion of the prescribing clinician (8%).

Treatment-naïve eyes (group 1) received four loading doses of faricimab at four weekly intervals, followed by a further injection at eight weeks. Treatment-resistant eyes were divided into two groups at the discretion of the clinician. The first group (group 2) followed the same regimen as the group 1 patients. The final group (group 3) was given a single faricimab injection and followed up again in eight weeks as pro re nata (PRN). The dosing schedule for all groups was then adjusted based on disease activity (Table 1) as per the ELHT protocol (Appendix 1 & 2).

**Table 1:** Definition of moderate to severe disease activity based on the ELHT protocol (Appendix 1 & 2). ELHT: East Lancashire Hospitals NHS Trust; OCT: optical coherence tomography; nAMD: neovascular age-related macular degeneration.

Criterion	Disease activity
Visual acuity	>5 letters decrease in best corrected visual acuity (BCVA) compared with the average BCVA from the last two visits OR >10 letters decrease in BCVA compared with the highest BCVA recorded over the previous two visits.
OCT	Increase in central retinal thickness (CRT) >50 μm compared with the average BCVA from the last two visits OR increase in CRT >75 μm compared with the lowest CRT recorded at the two previous visits.
Clinical examination	Presence of new macular haemorrhage due to nAMD activity OR significant nAMD activity at week 24 that requires immediate treatment.

Study design

The exclusion criteria included incomplete medical records or insufficient follow-up. Two patients were removed from the data (2%). The first patient relocated after one faricimab injection. The second patient died from other medical causes.

Collected data included patient demographics, treatment history, visual acuity (VA), number of faricimab injections, evidence of intraocular inflammation, and the presence of retinal fluid and fibrosis as determined by each investigator as demonstrated on optical coherence tomography (OCT). The Heidelberg Spectralis Optical Coherence Tomography (OCT) machine (Heidelberg Engineering, Heidelberg, Germany) was used to analyse the central retinal thickness (CRT) at each patient visit. It was deemed that the retinal anatomy had improved if there was at least 50 microns of retinal fluid resolution and/or an observed improvement in the size of the pigment epithelial detachment (PED). Retinal fluid included both intraretinal fluid (IRF) and subretinal fluid (SRF). On the first visit, all patients had a refracted best corrected visual acuity (BCVA) recorded by an optician using the Early Treatment Diabetic Retinopathy Study (ETDRS) and given a visual score in letters. At subsequent visits, the vision was recorded using the ETDRS letter score; this was not best corrected.

Statistical analysis

For statistical analysis, the paired t-test or Wilcoxon signed-rank test was employed to compare the VA (letters) and CRT (μm). For data collection, Excel (Microsoft Corporation, Redmond, WA) was used, with data analysis performed on Prism (Graphpad Software, Boston, MA). A p-value of <0.05 was considered statistically significant. Visits 1-4 were deemed the disease control phase. Visit 5 onwards was deemed the extension phase. For the eyes in groups 1 and 2 that did not complete the full loading dose of four faricimab injections at four-week intervals, the worst possible outcome was selected for these patients to reduce bias in the statistical analysis.

We have consulted with our institution’s ethics committee, and they have confirmed that ethical approval is not necessary for this type of retrospective analysis.

## Results

Introductory results and patient demographics

A total of 99 eyes (89 patients) were included in the study, giving a total of 350 faricimab injections. The total number of eyes in groups 1, 2, and 3 was 39, 32, and 28, respectively. The age of the patients ranged from 57 to 96 years (mean = 80.8 years). The patient demographics can be seen in Tables 2, 3.

**Table 2:** Patient demographics.

Variable	Groups	N (%)
Gender	Male	35 (35.4)
	Female	64 (64.6)
Eye	Left	59 (59.6)
	Right	40 (40.4)
Previous treatment	Aflibercept	19 (19.2)
	Ranibizumab	48 (48.5)
	Brolucizumab	19 (19.2)
	Treatment-naïve	39 (39.4)

**Table 3:** Prior ophthalmic diagnosis.

Prior ophthalmic diagnosis	Group	N (%)
Blepharitis	1	2 (5.1)
	2 & 3	2 (3.3)
Cataracts	1	7 (18.0)
	2 & 3	13 (21.7)
Glaucoma	1	0 (0.0)
	2 & 3	8 (13.3)
Phacoemulsification surgery	1	5 (12.8)
	2 & 3	4 (6.7)
Uveitis	1	0 (0.0)
	2 & 3	1 (1.7)

A total of 10 patients received bilateral treatment (20 eyes). The average number of injections given to group 1 and 2 eyes was four (range = 1-6 injections). The average number of injections given to group 3 eyes was three (range = 1-3 injections). Groups 2 and 3 patients had received treatment with previous anti-VEGF medications. A total of 24 eyes had between one and 10 previous anti-VEGF injections, 21 eyes had between 11 and 20 previous anti-VEGF injections, and 15 eyes had had more than 20 prior anti-VEGF injections.

**Figure 1:**
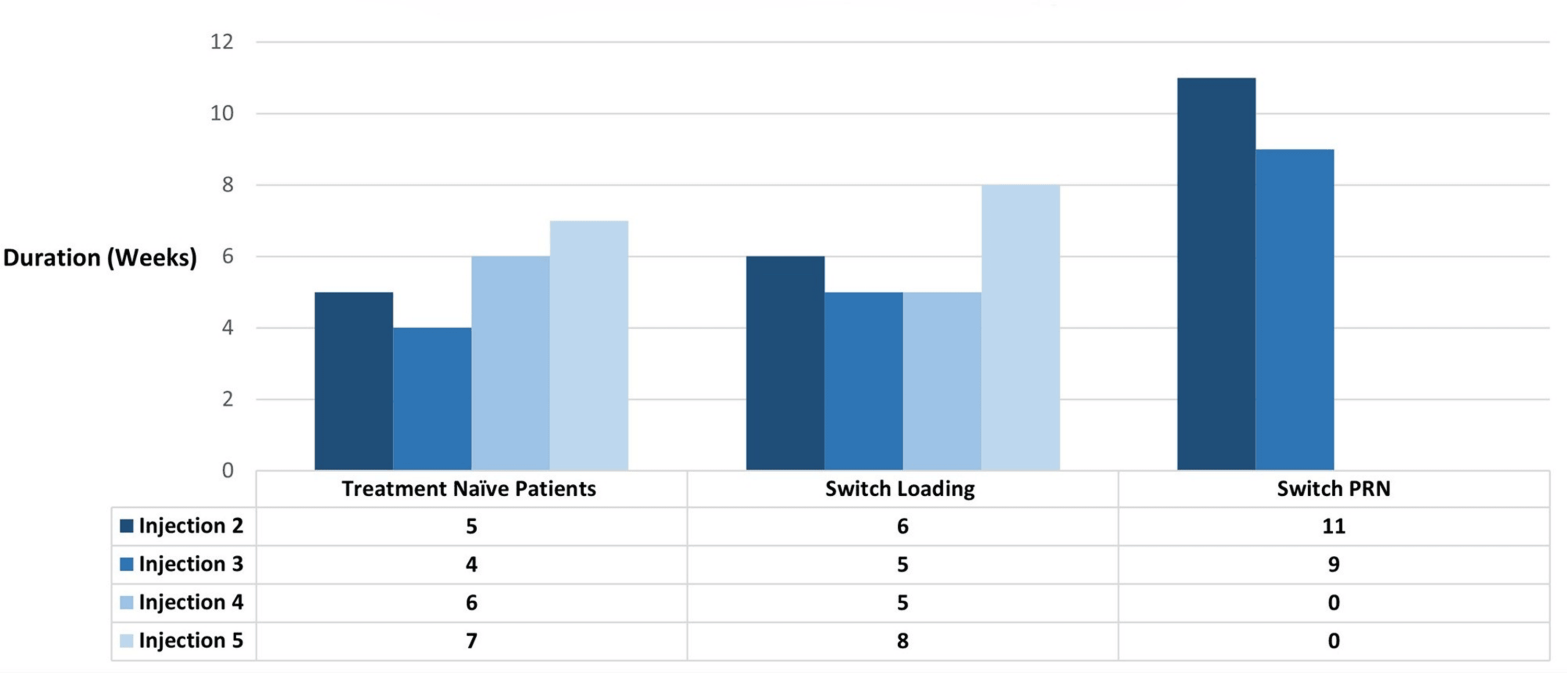
Mean number of weeks between faricimab injections for each study groups. PRN: pro re nata.

Eyes in group 1 had a mean treatment interval of 5.5 weeks (range = 4-7 weeks). Group 2 had a mean treatment interval of six weeks (range = 5-8 weeks), and group 3 had a mean treatment interval of 10 weeks (range = 9-11 weeks) (Figure 1).

Results summary

A summary of the efficacy data is presented in Table 4.

**Table 4:** Efficacy data for all groups of patients after receiving faricimab treatment in the study period. SEM: standard error of the mean; ETDRS: Early Treatment Diabetic Retinopathy Study; CRT: central retinal thickness; PRN: pro re nata.

Treatment efficacy per group (N)	Variable	Pre-treatment mean (SEM)	Post-treatment mean (SEM)	Change (SEM)	P-value
Overall efficacy (N = 99)	ETDRS (letters)	53.98 (1.38)	54.40 (1.88)	0.42 (1.43)	0.768
	CRT (µm)	408.25 (11.45)	315.80 (8.23)	-92.45 (11.84)	<0.0001
Efficacy in group 1: Treatment-naïve (N = 39)	ETDRS (letters)	55.58 (2.24)	52.38 (3.77)	-3.46 (3.00)	0.256
	CRT (µm)	426.82 (18.09)	298.74 (10.69)	-128.08 (15.65)	<0.0001
Efficacy in group 2: Treatment-resistant loading (N = 32)	ETDRS (letters)	56.38 (2.37)	55.59 (2.46)	-0.79 (2.13)	0.45
	CRT (µm)	421.75 (24.57)	306.28 (15.66)	-115.47 (25.99)	<0.0001
Efficacy in group 2: Treatment-resistant PRN (N = 28)	ETDRS (letters)	54.61 (2.73)	55.86 (3.06)	1.25 (1.19)	0.304
	CRT (µm)	366.96 (12.72)	350.43 (16.28)	-16.53 (11.55)	0.164

**Figure 2:**
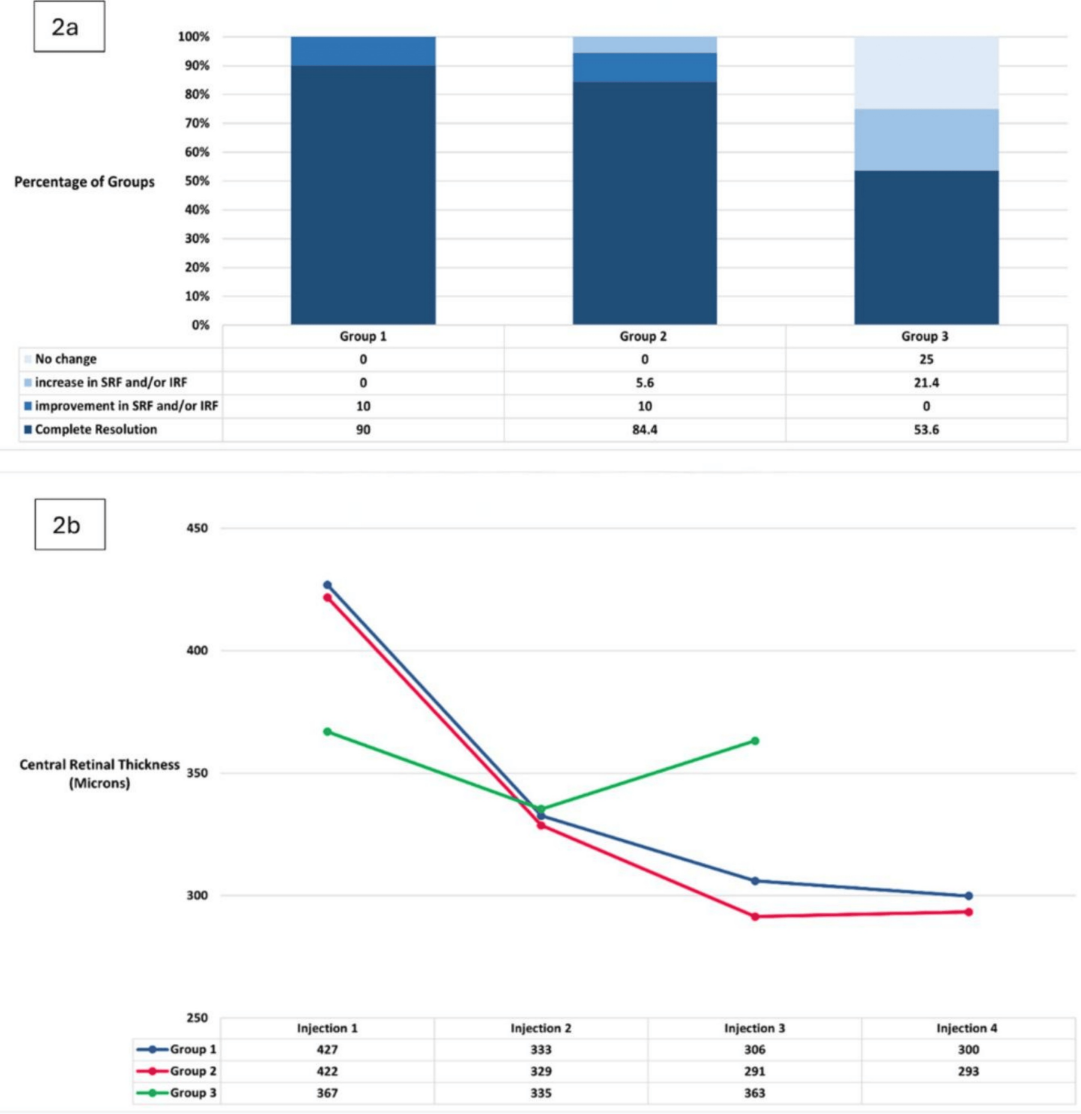
(a) Change in retinal fluid at final follow-up for each study group. (b) Change in mean CRT during faricimab treatment for each study group. SRF: subretinal fluid; IRF: intra-retinal fluid; CRT: central retinal thickness.

In group 1, 100% of eyes showed decreased retinal fluid after one injection, and at final follow-up, 90% had complete resolution of SRF and IRF. The remaining 10% showed improvement in SRF and/or IRF, with 74.4% stable at the last follow-up (average duration: 15 weeks). In group 2, 81.3% had decreased retinal fluid after one injection, with 84.4% achieving complete resolution at final follow-up. In 10%, there was improvement without total resolution, while 5.6% experienced an increase in fluid (range = +1 µm to +191 µm) (Figure 2A). In total, 75% were stable at the last follow-up (average duration: 17 weeks). In group 3, 64.2% had decreased fluid after one injection, and 53.6% achieved complete resolution at final follow-up. In 25%, there was no change, and 21.4% experienced an increase in fluid (range = +1 µm to +146 µm) (Figure 2A). In total, 46.4% were stable at the last follow-up (average duration: 13 weeks).

After faricimab treatment, group 1 had a mean CRT reduction of -128.08 µm (range = -6 µm to -364 µm) (p < 0.001, 95% CI: -96.39 to -159.76) (Figure 2B). Group 2 had a mean reduction of -115.47 µm (range = -4 µm to -608 µm) (p < 0.001, 95% CI: -608 to -91) (Figure 2B). In group 3, the mean reduction was -16.53 µm (range = -1 µm to -201 µm) (p = 0.164, 95% CI: 7.15 to -40.22) (Figure 2B).

The mean visual acuity for group 1 at the start was 55 ETDRS (range = 25-75 letters), with a post-treatment mean of 53 (range = 3-80 letters), resulting in a decrease of -3 ETDRS (p = 0.256, 95% CI: -9.53 to 2.61). In group 2, the mean pre-treatment acuity was 56 ETDRS (range = 20-77 letters), with no change post treatment (56, range = 20-77 letters) (p = 0.45, 95% CI: 0.102 to 8.77). For group 3, the mean starting acuity was 55 ETDRS (range = 25-77 letters), improving to 56 post treatment (range = 20-77 letters), showing an increase of 1 ETDRS (p = 0.304, 95% CI: -1.20 to 3.70).

Safety

From group 1, one patient developed bilateral intermediate uveitis after their second injection of faricimab (2%). The patient was a 79-year-old female with a history of bilateral pseudophakia. She presented with anterior chamber cells, vitritis, and vitreous snowballs that were treated with a course of both oral and topical steroids. The vision before faricimab was 70 ETDRS and 30 ETDRS in the left and right eyes, respectively. After receiving treatment, the patient’s vision was 75 ETDRS and 20 ETDRS in the left and right eyes, respectively. No patients in groups 2 or 3 developed intraocular inflammation during the study. There were no cases of endophthalmitis, retinal vasculitis, retinal artery occlusions, or RPE tears during the study period.

## Discussion

Faricimab was introduced at ELHT in November 2022. We began treating both treatment-naïve and treatment-resistant patients, which has allowed us to develop a trust protocol for the use of faricimab (Appendix 1 and 2).

After initial implementation, all treatment-resistant patients were treated PRN. However, an increase in CRT led to amending the ELHT protocol, dividing these patients into two groups: group 2, with moderate to severe disease activity, and group 3, with mild disease. Groups 1 and 2 received four loading doses of faricimab at four-week intervals, followed by an eight-week follow-up. If there was complete resolution of IRF and/or SRF, treatment intervals could be extended by four weeks, up to a maximum of 16 weeks. If disease activity persisted, intervals were not extended. Group 3 received a single faricimab injection and followed up in eight weeks PRN.

Dosing schedule and structural retinal changes

Our data indicate that the time between injections in groups 1 and 2 averaged four to six weeks (Figure 1), deviating from our ELHT faricimab protocol and highlighting challenges in adhering to a strict regimen in real-world settings. Nevertheless, group 1 patients consistently reduced CRT with each injection, with the most significant improvements after the first two injections (Figure 2B). Group 2 also saw CRT improvement after the first three injections, but the fourth injection resulted in an average increase of +3 µm (Figure 2B). This may be due to these patients' prior treatments and persistent disease. On average, group 2 received their fourth injection after five weeks, which could explain the CRT increase.

Group 3 patients were treated PRN, with injection intervals averaging seven to 11 weeks (Figure 1). Between the 2nd and 3rd faricimab injections (average interval of nine weeks), CRT increased (Figure 2B). This indicates that extended periods without loading doses in difficult-to-treat patients did not yield positive outcomes.

CRT and treatment-naïve patients

Group 1 eyes had a mean CRT reduction of -127.1 µm (Table 3), similar to the -137 µm reduction in the TENAYA and LUCERNE trials, which followed patients for about two years [6,7]. We hope our results will continue to align with these trials as more doses of faricimab are administered. While the manufacturer trials do not fully represent real-world scenarios, it is reassuring that our CRT improvement is comparable [6,7]. Another real-world study of 40 naïve eyes found baseline CRT of 214 ± 98 µm, decreasing significantly to 192 ± 89 µm at week 16 (P < 0.01) [8]. Notably, 10% of our treatment-naïve eyes did not complete the full course of four loading doses due to reasons such as sterile intraocular inflammation, patient non-attendance, and blepharitis. This deviation may have impacted our results.

CRT and treatment-resistant patients

Groups 2 and 3 eyes had a mean reduction in CRT of -115.47 µm and -16.53 µm, respectively, at the final follow-up appointment (Table 3). One real-world faricimab study looking at 190 treatment-resistant eyes in the United States found that CRT improved from 312 ± 87 μm to 287 ± 71 μm (P < 0.0001) [9,10]. A second study compared two groups: the first group was switched to faricimab, and the second control group remained on their current anti-VEGF treatment [8]. The results found that 39.3% of patients had a resolution of retinal fluid on OCT in the faricimab group in comparison to 7.4% in the control group (p = 0.004) [9]. These studies agree with our results that faricimab has a positive effect on the CRT of treatment-resistant patients.

SRF and IRF

At the final follow-up, groups 1, 2, and 3 achieved complete resolution of retinal fluid (SRF and/or IRF) of 90%, 84.4%, and 53.6%, respectively. This aligns with a real-world study of treatment-naïve patients, which reported 75% resolution for SRF and 66.67% for IRF [10]. Groups 1 and 2, receiving four loading doses of faricimab, outperformed group 3, which followed a PRN regimen (Figure 2A). Notably, even group 2, despite being treatment-resistant, had better resolution than group 3. Our data suggest that treatment-resistant patients should start with a loading dose regimen unless contraindicated, supporting previous findings of better visual outcomes with treat and extend compared to PRN regimens in nAMD patients [11,12].

Visual acuity

Our study did not show significant improvement in VA (Table 3). For group 1 eyes, the mean starting vision was 55 ETDRS letters, ending at 53 ETDRS (p = 0.256, 95% CI: -9.53 to 2.61). Group 2 had a mean starting vision of 56 ETDRS, remaining at 56 ETDRS (p = 0.256, 95% CI: -9.53 to 2.61). Group 3's mean starting vision was 55 ETDRS, ending at 56 ETDRS (p = 0.304, 95% CI: -1.20 to 3.70). While we would not expect significant VA increases in treatment-resistant patients (groups 2 and 3) due to long-term retinal changes, we anticipated improvements in treatment-naïve patients. Other real-world studies reported vision improvements in both naïve and resistant patients. One study noted a baseline VA of 0.33 ± 0.41 Logmar, improving to 0.22 ± 0.36 Logmar at week 16 (p < 0.01) [10]. A second study found that 35.7% of eyes treated with faricimab gained two or more lines of VA by the end of the four-month period [8]. For participants with incomplete data, we assigned the worst possible visual outcome to avoid sample bias.

Our study, being a real-world trial rather than a controlled one, may have influenced the VA outcomes. Each patient had their refracted BCVA assessed by an optician at the first appointment to ensure they met the NICE criteria of 6/96 vision or better for anti-VEGF eligibility. However, subsequent visits do not include BCVA assessments due to clinic demands; patients read from the ETDRS chart with support from clinic staff. This inconsistency in VA recording may explain the negligible change in VA compared to the significant CRT changes observed. Additionally, poor patient adherence to rigid treatment intervals is a challenge in real-world settings [13]. A large prospective study of 1,291 nAMD patients found similar results. They found that in patients treated with anti-VEGF therapies, delayed follow-up led to a significant decrease in VA that did not recover despite retreatment and normalisation of CRT [14].

Safety

For any clinician, the risk of inflammation or infection post-intravitreal injections is always a concern. From our real-world data over the course of eight months, there were no cases of endophthalmitis, retinal vasculitis, or retinal artery occlusions. From group 1, one patient developed bilateral sterile intraocular inflammation. This gives a 2% rate of inflammation. Several studies looking at the rates of sterile intraocular inflammation after aflibercept and ranibizumab found that the rate ranged from 0.05% to 4.4% [15,16]. Our rate of inflammation falls within this range, suggesting that faricimab has a comparable safety profile [6,16].

Study limitations

The study's limitations are notable in several key areas. First, the sample size for treatment-naïve patients (N = 30) is relatively small, which may limit the generalisability of the findings for this group. Additionally, the treatment-resistant group (N = 60) is heterogeneous, with patients receiving different dosing schedules, which could introduce variability in the outcomes. While the study employs a real-world design, it lacks a control group, making it difficult to definitively attribute observed changes in visual acuity and retinal anatomy to faricimab alone. The absence of randomisation and the observational nature of the study also heighten the risk of selection bias, as patients were selected based on clinical criteria rather than random assignment. Furthermore, the reliance on OCT images to assess retinal fluid and disease activity may be subject to inter-observer variability, given that these assessments were determined by individual investigators. The follow-up duration, which extends up to eight months, may not be sufficient to capture long-term outcomes or adverse effects of faricimab treatment. Additionally, the exclusion of two patients due to relocation and death reduces the robustness of the dataset, potentially underrepresenting certain patient outcomes. Lastly, the decision to use the worst possible outcome for patients who did not complete the full loading dose could introduce bias, potentially skewing the results toward a more negative assessment of treatment efficacy.

## Conclusions

This real-world study highlights the efficacy and safety of faricimab in both treatment-naïve and treatment-resistant patients. Our results are promising and show that patients who have been resistant to an alternative anti-VEGF can have an improvement if switched to faricimab. Our study took place over a duration of eight months, and a longer-term study is required to analyse the outcomes at 12 and 24 months.

## Figures and Tables

**Figure 3:**
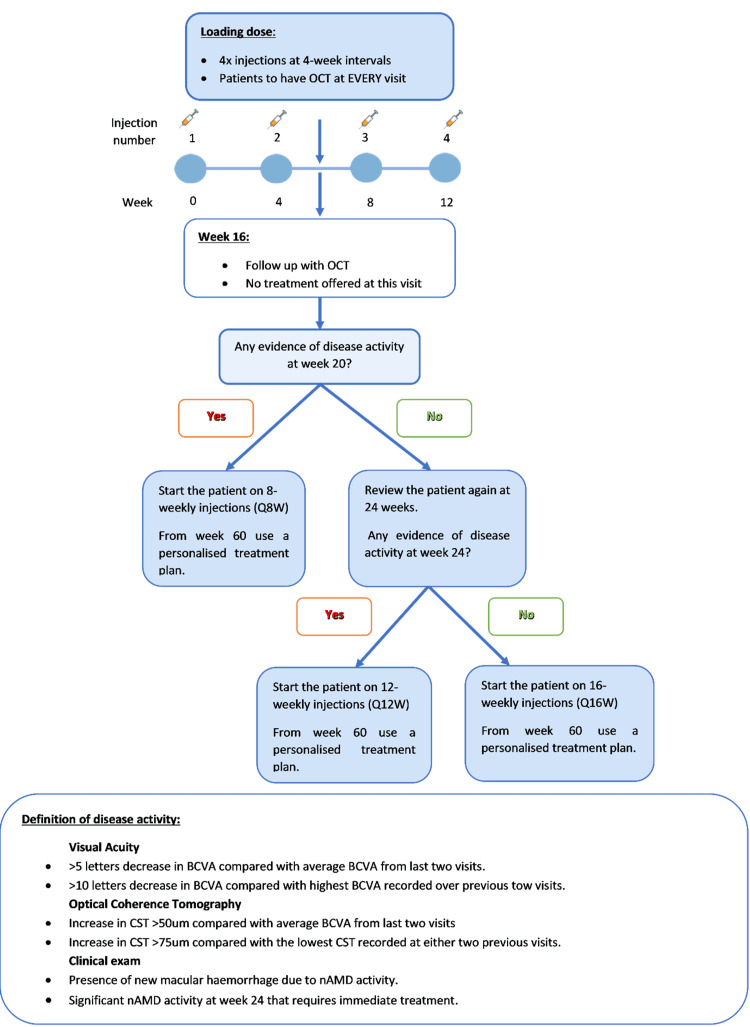
East Lancashire Hospitals NHS Trust – Faricimab protocol for treatment-naïve patients. OCT: optical coherence tomography; BCVA: best corrected visual acuity; CST: central subfield thickness; nAMD: neovascular age-related macular degeneration.

**Figure 4:**
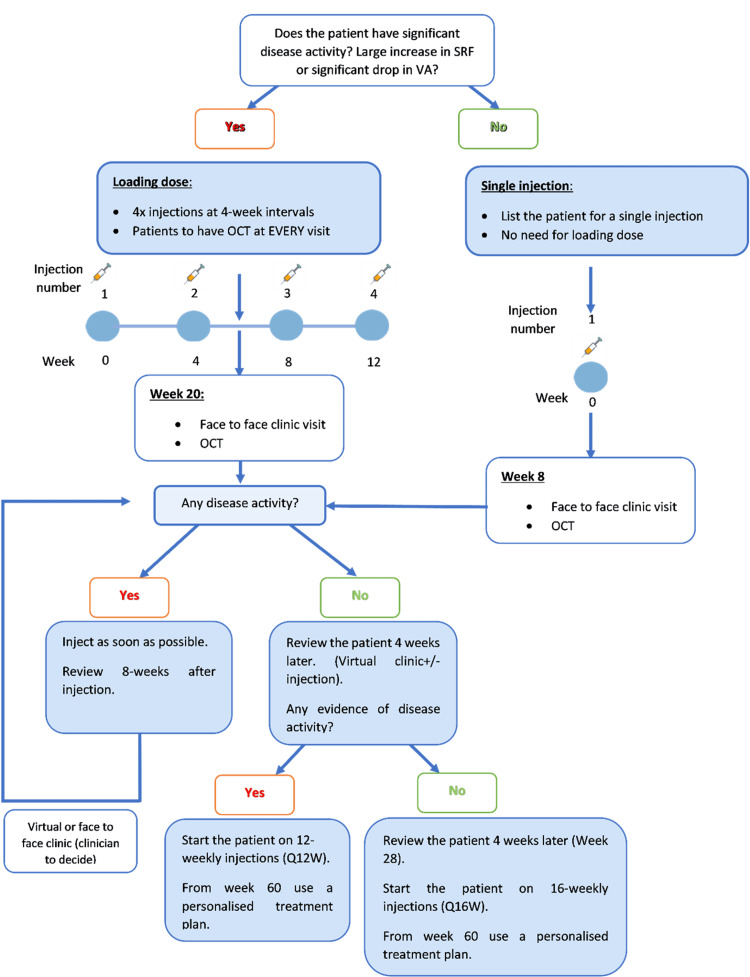
East Lancashire Hospitals NHS Trust – Faricimab protocol for treatment-resistant patients. SRF: subretinal fluid; OCT: optical coherence tomography; VA: visual acuity.

## Appendices

Appendix 1

Appendix 2
